# The COVID-19 pandemic and health workforce brain drain in Nigeria

**DOI:** 10.1186/s12939-022-01789-z

**Published:** 2022-12-05

**Authors:** Lukman Lawal, Abdulwahab Oluwatomisin Lawal, Opeyemi Pius Amosu, Abdulmujeeb Opeyemi Muhammad-Olodo, Nasir Abdulrasheed, Khalil-ur-Rahman Abdullah, Philemon Barnabas Kuza, Abdullahi Tunde Aborode, Yusuff Adebayo Adebisi, Ahmed Adeseye Kareem, Abdulwahab Aliu, Taiye Muhammed Elelu, Tonderai Murwira

**Affiliations:** 1 MCON Institute of Medical Research, Ilorin, Nigeria; 2 Faculty of Clinical Sciences, College of Health Sciences, University of Ilorin, Ilorin, Nigeria; 3 Faculty of Clinical Sciences, College of Medicine, University of Ibadan, Ibadan, Nigeria; 4 Healthy Africans Platform, Research and Development, Ibadan, Nigeria; 5 Department of Chemistry, Faculty of Physical Sciences, University of Ilorin, Ilorin, Nigeria; 6 Faculty of Pharmacy, University of Ibadan, Ibadan, Nigeria; 7 Department of Development Studies, Faculty of Humanities and Social Science, Lupane State University, 67, Mbizi Street, Mvurwi, Lupane, Zimbabwe

**Keywords:** COVID-19 pandemic, Medical brain-drain, Nurses, Pharmacists, Job satisfaction, Healthcare workers, Migration, Global skill partnership model, Nigeria, Africa

## Abstract

Over the years, the Nigerian healthcare workforce, including doctors, nurses, and pharmacists have always been known to emigrate to developed countries to practice. However, the recent dramatic increase in this trend is worrisome. There has been a mass emigration of Nigerian healthcare workers to developed countries during the COVID-19 pandemic. While the push factors have been found to include the inadequate provision of personal protective equipment, low monthly hazard allowance, and inconsistent payment of COVID-19 inducement allowance on top of worsening insecurity, the pull factors are higher salaries as well as a safe and healthy working environment. We also discuss how healthcare workers can be retained in Nigeria through increment in remunerations and prompt payment of allowances, and how the brain drain can be turned into a brain gain via the use of electronic data collection tools for Nigerian health workers abroad, implementation of the Bhagwati’s tax system, and establishment of a global skill partnership with developed countries.

**Graphical Abstract:**

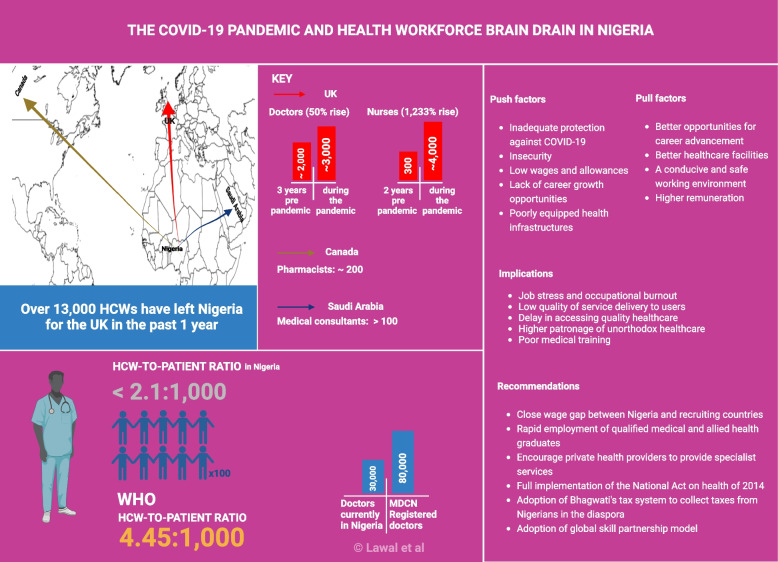

## Introduction

Brain-drain involves the migration of skilled workers out of their countries to more developed countries in search of a better standard of living in terms of better remuneration, better working conditions, and political stability [1]. Before the pandemic, the Nigerian health system faced poor funding, poor staff remuneration, and poor working conditions [2]. However, the emergence of the COVID-19 pandemic has further worsened those challenges and has created an atmosphere where Nigerian healthcare workers are further exhausted and dissatisfied with their jobs [3]. This has negatively affected healthcare delivery and medical education in Nigeria; hence, the need for urgent attention. This article aims to highlight the increased health workforce brain drain in Nigeria, the implications, and provide recommendations on not only stopping the brain drain but also converting it into a brain gain.

### The COVID-19 pandemic and health workforce brain drain in Nigeria

Prior to the pandemic, 88% (almost 9 in 10) of Nigerian doctors and about 50% of Nigerian nurses considered seeking job opportunities abroad, unless their working conditions improved [2, 4]. Unfortunately, the pandemic further strained the already fragile Nigerian healthcare system, resulting in serious negative impacts on its workforce [3].

Nigeria is reported to be the highest workforce exporting country in Africa. Topping her destination countries are the United Kingdom (UK), United States (US), Canada, Australia, and Saudi-Arabia [2]. A national statistical report published in August 2022 by the UK government revealed that 13,609 healthcare workers have left Nigeria for the UK between 2021 to 2022 [5]. This figure is second only to Indians at 42,966, while the Philippines is third with 11,021 healthcare workers.

**Fig. 1:**
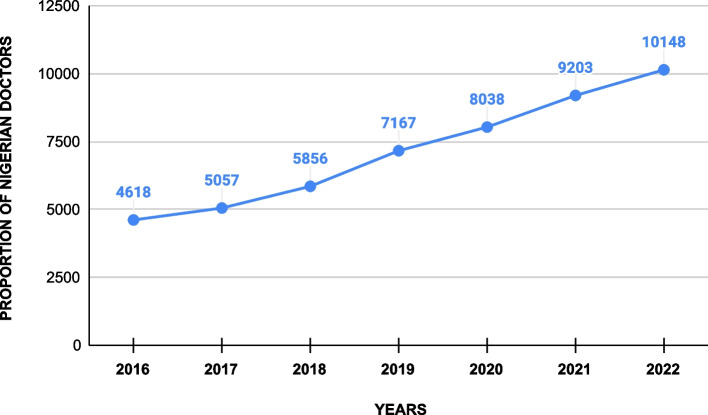
Number of Nigerian doctors on the GMC (UK) register before and during the COVID-19 pandemic

According to the General Medical Council of the UK register, over three years preceding the pandemic (January 2017 – December 2019), the total number of doctors that left Nigeria to practice in the UK was about 2,000 in comparison to around 3,000 recorded between January 2020 to September 2022, as illustrated in Fig. 1 [6], with many others currently writing or planning to write international licensing exams such as Professional Linguistics Assessment Board (PLAB) exam and the United States Medical Licensing Exam (USMLE). In addition, the Medical and Dental Consultants Association of Nigeria recently lamented that more than 100 medical consultants (specialists) and hundreds of junior doctors had left Nigeria for Saudi Arabia and others between 2020 to 2022 [7, 8].

**Fig. 2:**
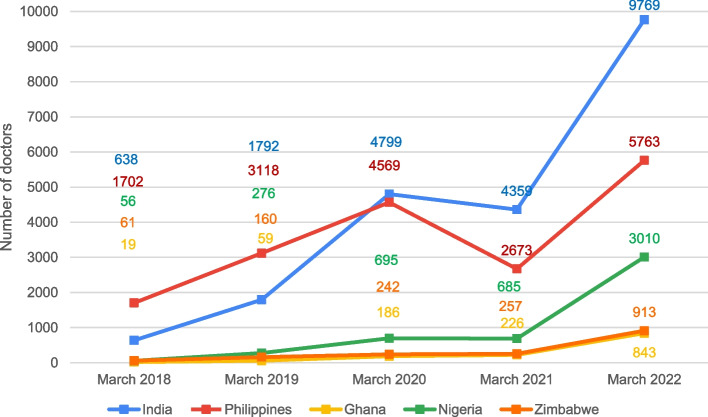
Top five countries’ nurses and midwives joining the UK workforce between March 2018 to March 2022

Similarly, according to the Nursing and Midwifery Council of the UK data, pre-COVID-19 pandemic, the proportion of Nigerian nurses and midwives immigrants increased from 56 in March 2018 to 276 in March 2019 compared to the pandemic period. This figure declined slightly from 695 in March 2020, to 685 in March 2021, possibly due to the transnational lockdown, then steeply rose in March 2022, to an all-time high of 3,010 (> 1000% increase), as shown in Fig. 2 [9].

In the same vein, the National Chairman, Association of Hospital and Administrative Pharmacists of Nigeria, reported that around 200 pharmacists have left Nigeria to practice abroad [10]. These call for concern and it is unsurprising that similar trends have also been widely reported in other low and low-middle-income countries such as Zimbabwe, South Africa, and Egypt to mention but a few [11, 12].

Major “push factors”, such as, inadequate protection against COVID-19, insecurity, and lack of death gratuity [13], in addition to pre-existing factors, like low wages and allowances (for example, monthly payment of hazard allowance of 5000 naira = US $11), lack of career growth opportunities, poorly equipped health infrastructures, etc. are the key issues needed to be addressed. The incidence of morbidity and mortality continues to rise among health workers as they continue to be in direct contact with numerous infectious cases. Over a thousand Nigerian healthcare workers had tested positive for the virus in less than a year with many mortalities [3]. This is a stark reflection of the 4% of the federal government budget allocated to healthcare. A far cry from the minimum 15% of budgetary allocation agreed upon by African leaders in the "2001 Abuja Declaration" [14].

### The COVID-19 pandemic and health workforce shortage in developed countries

Developed countries aren’t unaffected by the pandemic, with overwhelming COVID-19 cases and deaths. The shortfall of physicians in the US, for example, is estimated to grow to nearly 95,000 by 2025 [14]. In the efforts of the US government to bridge this gap, the US Foreign Mission was reported to have advised medical professionals with approved immigration petitions to contact its embassies or consulates for possible visa appointments, specifying a preference for those working to combat the COVID-19 pandemic.

Likewise, the UK government implemented a new ‘Health and Care Visa’ policy, aiming to make it faster and cheaper for international medical graduates to migrate to the UK to practice [14]. Other several EU countries also waived strict immigration regulations on foreign-trained health personnel to facilitate reinforcement of their workforce.

The pull factors that drive the intention of the Nigerian health workforce to leave their jobs for greener pastures abroad have been found to include better opportunities for career advancement, better healthcare facilities, a conducive working environment, and higher remuneration [2]. For instance, Nigerian Professors of Medicine in Saudi-Arabia on average are paid between 5 and 7 million naira ($12,138—$16,994) monthly compared with 420,000 naira ($1,019) received by their counterparts in Nigeria [8].

### Brain drain and healthcare delivery in Nigeria

Less than 50% (30,000) of the over 80,000 doctors registered with the Medical and Dental Council of Nigeria are currently practicing in the country. In a bid to facilitate universal health coverage, the world health organization recommends a minimum of 4.45 doctors, nurses, and midwives per 1,000 population; Nigeria has below 2.1 [15]. This critical shortage causes a delay in accessing quality healthcare, low usage of accredited health facilities, and higher patronage of unorthodox healthcare. These are key factors contributing to poor health indices of the nation [16].

Brain drain is also associated with job stress and occupational burnout, which affect health workers’ job performance, lead to low quality of service delivery to users, and fuels their intentions to leave their jobs [17]. Furthermore, the shortage of medical specialists has affected medical training in Nigeria, which translate to poor medical graduates and research output [18]. Having highlighted all these, universal health coverage, and other indicators of sustainable development goal three would be challenging to achieve by the projected year 2030 in Nigeria.

### Strategies to stem the tide

In light of the above findings, it is imperative for the relevant stakeholders to urgently take steps to retain its health workforce and turn the tide against brain drain. We recommend closing the wage gap between Nigeria and recruiting countries. To achieve this, the government should increase the health budget’s allocation from the current 4% to 15% and create a new funding mechanism for the healthcare sector. For instance, tax proceeds from harmful products such as alcohol and tobacco can be allocated to the health sector [14].

Also, there should be rapid employment of qualified medical and allied-health graduates to alleviate the shortage and create career advancement opportunities for the workforce. In addition, an enabling environment for private health providers to expand their capacity to provide specialist services should be encouraged, while ensuring there is minimal wage discrepancy between government workers and private employees to prevent a risk of internal brain-drain. Furthermore, the government should implement the recommendations of the Yayale Ahmed Presidential Committee on Health and the National Act of 2014, which aim to provide a framework for the regulation, development, and management of a national health system and set the standards for rendering health service in the federation [19].

Moreover, the Nigerian national policymakers on human resources for health migration should not prohibit migration. Instead, it should call for an ethical method of migration that allows Nigeria to collaborate with recruiting countries to ensure mutual benefits [20]. This can be achieved by designing an electronic database record for Nigerian healthcare workers in diaspora and creating a similar system as implemented in the Bhagwati's tax system, which enables the collection of taxes from emigrants to their country of origin, as practiced by the US and Cuba [21]. In addition, the government should create an enabling environment for the healthcare workers of Nigerian origin in the diaspora to bring capital, management, and skills back to Nigeria, as is done by India and China.

Proactively, the policymakers should consider adopting the global skill partnership model, a bilateral labor agreement between a country of origin and a country of destination. While the country of origin trains students in skills needed to meet their specific and immediate needs and that of the country of destination, the latter provides the required finance and facility and, in turn, receives skilled migrants [15]. Multinational collaborations between Nigeria and other foreign countries would help to foster bilateral relationships and encourage the development of a model which would help to improve the health systems for all, so long the model is well designed, implemented, financed, and monitored by the relevant stakeholders (governments, workers’ and employers’ unions, civil society, financial auditors, and other public institutions) [22].

## Conclusion

The health workforce brain drain in Nigeria is not a new phenomenon, but the current increasing trend is alarming. The worsening of the “push factors” and strengthening of the “pull factors” by the COVID-19 pandemic has resulted in the mass emigration of Nigerian health workers to developed countries. The negative impact on healthcare delivery and medical education in Nigeria is unprecedented. We recommend closing the gap in wage disparity between Nigeria and the recruiting countries and adopting the global partnership skills model, among others, to retain healthcare workers in Nigeria, stem the tide against brain drain, and strengthen the health system for all.

## Data Availability

Not applicable.
